# PRMT7: A Pivotal Arginine Methyltransferase in Stem Cells and Development

**DOI:** 10.1155/2021/6241600

**Published:** 2021-10-19

**Authors:** Bingyuan Wang, Mingrui Zhang, Zhiguo Liu, Yulian Mu, Kui Li

**Affiliations:** ^1^Institute of Animal Sciences, Chinese Academy of Agricultural Sciences, Beijing 100193, China; ^2^College of Animal Science and Technology, China Agricultural University, Beijing 100193, China; ^3^Agricultural Genomes Institute at Shenzhen, Chinese Academy of Agricultural Sciences, Shenzhen 518120, China

## Abstract

Protein arginine methylation is a posttranslational modification catalyzed by protein arginine methyltransferases (PRMTs), which play critical roles in many biological processes. To date, nine PRMT family members, namely, PRMT1, 2, 3, 4, 5, 6, 7, 8, and 9, have been identified in mammals. Among them, PRMT7 is a type III PRMT that can only catalyze the formation of monomethylarginine and plays pivotal roles in several kinds of stem cells. It has been reported that PRMT7 is closely associated with embryonic stem cells, induced pluripotent stem cells, muscle stem cells, and human cancer stem cells. PRMT7 deficiency or mutation led to severe developmental delay in mice and humans, which is possibly due to its crucial functions in stem cells. Here, we surveyed and summarized the studies on PRMT7 in stem cells and development in mice and humans and herein provide a discussion of the underlying molecular mechanisms. Furthermore, we also discuss the roles of PRMT7 in cancer, adipogenesis, male reproduction, cellular stress, and cellular senescence, as well as the future perspectives of PRMT7-related studies. Overall, PRMT7 mediates the proliferation and differentiation of stem cells. Deficiency or mutation of PRMT7 causes developmental delay, including defects in skeletal muscle, bone, adipose tissues, neuron, and male reproduction. A better understanding of the roles of PRMT7 in stem cells and development as well as the underlying mechanisms will provide information for the development of strategies for in-depth research of PRMT7 and stem cells as well as their applications in life sciences and medicine.

## 1. Introduction

Arginine methylation is a common posttranslational modification of proteins, playing an essential role in several biological processes, such as mRNA splicing, DNA repair, transcription regulation, and signal transduction [1–4]. In mammals, arginine methylation is catalyzed by protein arginine methyltransferases (PRMTs) through transferring methyl groups from S-adenosylmethionine to the specific arginine residues of protein substrates [5]. Currently, nine PRMTs have been characterized and classified into type I, type II, and type III PRMTs according to the type of catalyzed arginine methylation reaction. Among these three types of PRMTs, type I PRMTs which contain PRMT1, PRMT2, PRMT3, PRMT4/CARM1 (coactivator associated arginine methyltransferase 1), PRMT6, and PRMT8 catalyze the formation of monomethylarginine and asymmetric dimethylarginine. Type II PRMTs which contain PRMT5 and PRMT9 catalyze the formation of monomethylarginine and symmetric dimethylarginine, whereas type III PRMT7 can only catalyze the formation of monomethylarginine [6–8].

Most PRMTs tend to methylate glycine- and arginine-rich motifs in proteins [9]. For example, PRMT1, PRMT3, and PRMT6 target glycine- and arginine-rich motifs, which are related to the regulation of nucleic acid and protein interactions [9–13]. PRMT4 (also known as CARM1) methylates proline-, glycine-, and methionine-rich motifs located on splicing and transcription elongation factors, which can affect alternative splicing [14]. PRMT5 can methylate glycine- and arginine-rich motifs as well as proline-, glycine-, and methionine-rich motifs [14, 15]. However, PRMT7 can specifically methylate a motif in lysine- and arginine-rich regions, which contains a pair of arginine residues separated by one residue [16, 17]. To date, the known substrates methylated by PRMT7 include the core histones (H2A, H2B, H3, and H4), the Wnt signaling molecule Dishevelled3, and the transcription factor C/EBP-*β*, implying that PRMT7 could be involved in a wide range of cellular processes both in normal and disease states [8, 18–20]. In this review, we mainly focus on the roles of PRMT7 in stem cells and in murine and human development. By summarizing the functions of PRMT7 in mouse embryonic stem cells (ESCs), induced pluripotent stem cells (iPSCs), muscle stem cells (MuSCs), and cancer stem cells, we found that PRMT7 was crucial for the pluripotency, proliferation, and differentiation of stem cells. By summarizing the phenotypes of PRMT7 deficiency or mutation in mice and humans, we concluded that PRMT7 was important for the development of skeletal muscle, neurons, bone, adipose tissues, and male reproduction. This review is aimed at providing information for the development of strategies for the in-depth research of PRMT7 and stem cells as well as their applications in life sciences and medicine.

## 2. The Roles of PRMT7 in Stem Cells

### 2.1. The Role of PRMT7 in ESCs

Mouse ESCs are the main type of pluripotent cells that have attracted considerable research interest since they were defined [21–23]. However, the mechanisms underlying their functions are still being explored. In 2008, given the pluripotent characteristics of mouse ESCs and embryonic germ cells, Buhr et al. performed nuclear proteome analysis to identify the nuclear proteins potentially associated with pluripotency. Finally, they discovered a new protein, PRMT7, which behaved as a candidate protein related to pluripotency [24].

The further functional study revealed that PRMT7 depletion in mouse ESCs using shRNA led to spontaneous differentiation and G1 arrest of the cell cycle, suggesting the crucial role of PRMT7 in stemness maintenance [25]. The mechanistic analysis in this study discovered that PRMT7 played an inhibitory role in the expression of miR-24-2 gene-encoding miR-24-3p and miR-24-2-5p. Overexpression of miR-24-3p/miR-24-2-5p can induce ESC differentiation similar with PRMT7 depletion. Moreover, inhibition of miR-24-3p/miR-24-2-5p can reverse ESC differentiation induced by PRMT7 depletion. In addition, as the key pluripotency factors, Oct4, Nanog, Klf4, and c-Myc were also detected [26–29]. It turned out that the expression of Oct4, Nanog, Klf4, and c-Myc was repressed after PRMT7 depletion or miR-24-3p/miR-24-2-5p overexpression. Overall, these results demonstrate that PRMT7 is crucial for the maintenance of ESC stemness by repressing miR-24-3p and miR-24-2-5p as well as pluripotency factors Oct4, Nanog, Klf4, and c-Myc.

Later, to gain deeper insight into the molecular mechanism underlying how PRMT7 regulated the pluripotency of mouse ESCs, additional miRNAs were identified to be involved in the maintenance of mouse ESC stemness. As a result, miR-221 gene-encoding miR-221-3p and miR-221-5p were discovered as negative regulators for the maintenance of mouse ESC stemness through their direct repression by PRMT7 [30]. The study demonstrated that miR-221-3p and miR-221-5p not only downregulated the expression of Oct4, Nanog, and Sox2 but also induced spontaneous differentiation of mouse ESCs, which is similar to the results from PRMT7 depletion experiments. Further, inhibition of miR-221-3p and miR-221-5p can block PRMT7 depletion-induced differentiation of mouse ESCs. Therefore, these results demonstrate that besides miR-24-3p and miR-24-2-5p, PRMT7 also mediates the repression of miR-221-3p and miR-221-5p which can target and downregulate Oct4, Nanog, and Sox2, thus playing crucial roles in maintaining the stemness of mouse ESCs.

Notably, both mouse mature oocytes and ESCs can reprogram somatic cells to a pluripotent state through somatic cell nuclear transfer and cell fusion, respectively [31–35]. Therefore, to survey reprogramming factors, a proteome analysis of mouse mature oocytes and ESCs was performed. PRMT7 was identified as a candidate reprogramming factor common to mouse mature oocytes and ESCs [36]. To better determine the role of PRMT7 in inducing pluripotency, each one of the four general reprogramming factors, namely, Oct4, Sox2, Klf4, and c-Myc, used to generate mouse iPSCs was replaced by PRMT7. Results showed that PRMT7 can replace Sox2 to establish iPSCs in combination with Oct4, Klf4, and c-Myc [37, 38]. Taken together, these findings suggest that PRMT7 is a pivotal regulator of the pluripotency of both mouse ESCs and iPSCs.

### 2.2. The Role of PRMT7 in MuSCs

MuSCs are responsible for muscle growth and repair by sustained self-renewal and differentiation [39, 40]. vanLieshout et al. discovered that PRMT1, PRMT4, PRMT5, and PRMT7 were the most abundantly expressed PRMTs in human muscle, implying the potential role of these four PRMTs in muscle development [41].

Thereafter, PRMT5 was first studied in skeletal MuSCs. The results demonstrated that PRMT5 could control the proliferation of adult mouse MuSCs by directly silencing the cell cycle inhibitor p21 [42]. Similarly, Blanc et al. found that PRMT7-deficient MuSCs also underwent cell cycle arrest with increased p21 levels [43]. Further analysis revealed that PRMT7 was preferentially expressed and colocalized with Pax7, a marker of quiescent skeletal muscle stem cells [44, 45], in the nuclei of MuSCs. In the process of differentiation, when the majority of wild-type MuSCs underwent differentiation, PRMT7^−/−^ MuSCs displayed defects in differentiation. RNA sequencing analysis revealed that PRMT7 was a cell cycle regulator. Subsequently, the immunoblotting assay confirmed the elevated levels of the cell cycle inhibitor p21 in PRMT7^−/−^ MuSCs. As a known repressor of p21, the expression of DNMT3b usually is inversely correlated with that of p21 [46]. So, the expression of DNMT3b in PRMT7-deficient MuSCs was assessed, which showed that PRMT7 deficiency reduced the expression of DNMT3b in MuSCs. In addition, given that elevated expression of p21 is a cellular senescence marker [47, 48], further experiments revealed that PRMT7-deficient MuSCs or myoblast cell line existed senescence. Restoring DNMT3b expression can rescue the senescence and decline p21 expression induced by PRMT7 deficiency. These discoveries demonstrate that PRMT7 plays a vital role in skeletal muscle development by regulating the proliferation and differentiation of MuSCs through the DNMT3b/p21 pathway.

### 2.3. PRMT7 in Human Cancer and Cancer Stem Cells

Through a meta-analysis of gene expression in more than 120 breast tumors, PRMT7 was identified as a potential metastasis-promoting gene in breast cancer [49]. In cancer, epithelial-to-mesenchymal transition (EMT) could cause metastasis, and loss of E-cadherin expression is a hallmark of EMT [50, 51]. PRMT7 was reported to be highly expressed in breast carcinoma cells in which it inhibited E-cadherin expression, consequently mediating breast cancer metastasis. Silencing PRMT7 can restore E-cadherin expression [52]. Accordingly, PRMT7 was also overexpressed in breast cancer tissues and invasive breast cancer cells. Reduced expression of PRMT7 inhibited breast cancer cell invasion both *in vitro* and *in vivo*. Further mechanistic study revealed that PRMT7 promoted breast cancer cell invasion by regulating MMP9 (matrix metalloproteinase 9) which is a famous mediator of breast cancer metastasis [53]. Moreover, as a methyltransferase, PRMT7 mediated E-cadherin expression through PRMT7 automethylation, which consequently triggered EMT [54]. In addition to direct inhibition and automethylation, a recent study discovered that PRMT7 promoted the metastasis of human breast cancer by methylating the arginine of SHANK2 (SH3 and multiple ankyrin repeat domains 2), consequently activating endosomal FAK (focal adhesion kinase) signaling [55], which led to cancer cell growth to a malignant phenotype [56]. These findings demonstrate that PRMT7 plays a pivotal role in breast cancer metastasis through downregulating E-cadherin, upregulating MMP9, and activating FAK. Targeting PRMT7 expression or enzyme activity could be a therapeutic strategy for human breast cancer.

Similar function of PRMT7 has also been reported in human non-small-cell lung cancer (NSCLC). An online database analysis indicated that lung cancer tissues exhibited higher PRMT7 expression than healthy tissues [57]. The functional study demonstrated that overexpression of PRMT7 promoted NSCLC cell invasion and colony formations. The coimmunoprecipitation assay against PRMT7 combined with mass spectrometry analysis with two types of NSCLC cell lines, namely, A549 and SPC-A1, discovered 19 shared target proteins. Among these in-common targets, HASP5 (heat shock protein 5) and EEF2 (eukaryotic translation elongation factor 2) were validated interacting with PRMT7. Both HASP5 knockdown and EEF2 knockdown can significantly restore PRMT7 overexpression-induced NSCLC invasion, indicating that PRMT7 promoted metastasis in NSCLC likely through interacting with HSPA5 and EEF2, which provides information on the mechanism of lung cancer metastasis and a candidate target gene in lung cancer. In addition to breast cancer and lung cancer, it was recently found that PRMT7 expression was increased in clear cell renal cell carcinoma tissues as well. There was a close correlation between increased PRMT7 expression and poor prognosis of clear cell renal cell carcinoma [58]. Further assays discovered that PRMT7 promoted the proliferation of renal cell carcinoma both *in vitro* and *in vivo*. Mechanistically, PRMT7 upregulated c-Myc expression, which was blocked by knocking down *β*-catenin. PRMT7 kept *β*-catenin stabilization by methylating *β*-catenin and thus inhibiting its ubiquitination as well as degradation. Overall, PRMT7 also serves as an oncogene in renal cell carcinoma via the *β*-catenin/c-Myc pathway.

As abovementioned, PRMT7 maintained mouse ESC stemness by repressing miR-24-2 gene-encoding miR-24-3p and miR-24-2-5p [25]. We noticed that miR-24-2 was also associated with human tumorigenesis, but it had both tumor suppressive and oncogenic effects on different cancer types. For example, it promoted the development of gastric cancer and esophageal squamous cell carcinoma [59, 60] but decreased the tumorigenicity of MCF-7 breast cancer cells and served as a negative biomarker of colorectal cancer [61, 62]. Liver cancer as one of the most common cancers can cause a high mortality rate, which is mainly attributed to liver cancer stem cells (hLCSCs). The hLCSCs exhibit the properties of stem cells, namely, self-renewal and differentiation, which have been considered a therapeutic target of liver cancer [63]. Therefore, Wang and colleagues explored the roles and relationship between miR-24-2 and PRMT7 in hLCSCs [64]. Similar to the findings in mouse ESCs, miR-24-2 also targeted the 3′ untranslated region of PRMT7 in hLCSCs, which inhibited the expression of PRMT7. Moreover, miR-24-2 promoted the proliferation ability and tumorigenic ability of hLCSCs *in vitro* and *in vivo*, respectively, possibly through negatively regulating PRMT7. These results suggested that Prmt7 might be negatively correlated with the proliferation of hLCSCs, implying the distinctive role of Prmt7 regulated by miR-24-2 in liver cancer.

## 3. The Roles of PRMT7 in Development

### 3.1. Phenotypes of PRMT7 Deficiency in Mice and Humans

An optimal way to better understand the roles of a gene at an individual level is to study the effects of a gene's loss-of-function. In 2015, Ying et al. generated PRMT7^−/−^ mice with a C57/bl6-129sv background. However, most PRMT7^−/−^ mice died within 5~10 days after birth, indicating the essential role of PRMT7 in normal development [65]. Moreover, when PRMT7 was deleted in C57BL6/J mice, these mice were subviable as only 45% of the expected number of PRMT7^−/−^ pups were obtained at postnatal day 14. At postnatal day 10, the PRMT7^−/−^ pups displayed reduced body size and weight, as well as skeletal defects. The adult PRMT7^−/−^ mice showed limb bone anomalies, reduced length, and increased fat mass [66]. However, Blanc and colleagues revealed that PRMT7^−/−^ mice were viable, but smaller at birth than wild-type mice. Although the weight difference was sustained only up to 3 months of age, necropsy of 8-month-old PRMT7^−/−^ mice exhibited less skeletal muscle mass and more epididymal fat. Muscles derived from PRMT7^−/−^ mice displayed decreased oxidative metabolism [67]. In addition, Lee and colleagues reported that PRMT7^−/−^ mice showed hyperactivity as well as social interaction defects [68]. These findings demonstrate that PRMT7^−/−^ mice have defects in bone and skeletal muscle mass, delayed or impaired neuronal development, increased adipogenesis, and possibly also reduced male fertility.

Regarding the phenotypes in PRMT7-mutated humans, Akawi and colleagues found recessive causation of PRMT7 in 4125 families following cosegregation studies and performed a more in-depth clinical assessment. After identifying 6 affected females from 3 families with PRMT7 mutation, they observed that the associated clinical phenotype was similar to the phenotype of pseudohypoparathyroidism, namely, Albright hereditary osteodystrophy. The individuals exhibited obesity, mild intellectual disability, symmetrically shortened digits, metatarsals, and posterior metacarpals [66]. Later, a male child was confirmed as PRMT7 null in function, and the child exhibited severe intellectual disability, seizures, short stature, microcephaly, facial dysmorphism, brachydactyly, and cryptorchidism [69]. In the same year, Agolini and colleagues reported 3 additional patients with PRMT7 mutation. The phenotype they exhibited was defined as a novel intellectual disability syndrome, namely, SBIDDS (short stature, brachydactyly, intellectual developmental disability, and seizures) [70]. Whole exome sequencing analysis identified a homozygous private nonsense change in exon 4 and a sibling-shared homozygous missense variant in exon 13 of PRMT7. Thereafter, another patient with SBIDDS was reported additionally displaying psychomotor delay and hearing loss, and two PRMT7 mutations were identified through the patient's whole exome sequencing [71]. In addition, Birnbaum et al. investigated PRMT7-related syndrome at prenatal and postnatal stages in two male siblings who were homozygous for a mutation in PRMT7 [72]. Both of them displayed intrauterine growth restriction due to the defects in long bone. The first child was terminated, and autopsy findings showed eye tumor, whereas the second child exhibited postnatal growth restriction, skeletal involvement, hypotonia, sensorineural hearing loss, strabismus, genitourinary, and global developmental delay, which provided additional pathological and clinical data and expanded the phenotypes of PRMT7 mutations [72]. These findings indicate that PRMT7 mutations could lead to severe developmental defects in skeletal muscle, neurons, bones, and possibly male reproduction, similar to the phenotypes of PRMT7^−/−^ mice.

### 3.2. PRMT7 in Skeletal Muscle Development

We have introduced the role of PRMT7 in MuSCs and the skeletal muscle-related phenotypes of PRMT7 mutations in mice and humans. Here, we further summarize the studies on the role of PRMT7 in skeletal muscle development. Through qRT-PCR analysis of adult mouse tissues, including the liver, small intestine, stomach, lung, kidney, spleen, pancreas, heart, skeletal muscle, and white and brown adipose tissues, it is shown that PRMT7 was most abundant in the skeletal muscle. Furthermore, with the qRT-PCR assay and immunofluorescence analysis, it is found that PRMT7 deficiency caused a significantly reduced expression of oxidative fiber markers MyhI and MyhIIa, as well as a substantially increased expression of glycolytic fiber markers MyhIIx and MyhIIb, compared with the wild type [67]. Moreover, PRMT7 expression was lower in obese mice compared with normal mice. Mice with PRMT7 deficiency exhibited less skeletal muscle mass, and PRMT7^−/−^ muscle displayed decreased oxidative metabolism and reduced expression of oxidative metabolism-related genes, such as PGC-1*α*. Mechanistic studies using PRMT7^−/−^ myoblasts revealed that PRMT7 regulated oxidative metabolism in muscles by activating the p38MAPK/ATF2/PGC-1a pathway. In addition, PRMT7 was also involved in myoblast differentiation since depletion of PRMT7 in myoblasts impaired cell cycle withdrawal and subsequent myogenic differentiation [73]. The underlying mechanism involved PRMT7-mediated methylation of p38MAPK on arginine 70 to activate p38MAPK, which further enhanced MyoD activity to mediate myoblast differentiation [73, 74]. Furthermore, PRMT7 was found to serve as downstream of apigenin (a natural flavone abundant in many plant-derived foods, such as parsley and celery), thus enhancing skeletal muscle hypertrophy and myoblast differentiation by regulating the PGC-1*α*/GPR56 pathway and the p38MAPK-myoD pathway, respectively [75, 76]. Taking these together, PRMT7 participates in skeletal muscle development by involving in skeletal muscle oxidative metabolism, myoblast differentiation, and hypertrophy through p38MAPK/ATF2/PGC-1a, p38MAPK/myoD, and PGC-1*α*/GPR56 pathways, respectively.

### 3.3. PRMT7 in Neuronal Development

Notably, patients with PRMT7 mutations displayed neuron-deficient phenotypes, such as cognitive deficits, brain abnormalities, and seizures, suggesting PRMT7 may play crucial roles in neural development [70]. Dhar and colleagues once used pluripotent human embryonal carcinoma cell line NTERA-2 clone D1 cells to investigate the role of MLL4 (mixed-lineage leukemia 4) in neuronal differentiation. They found that PRMT7, but not PRMT5, antagonized MLL4-mediated neuronal differentiation by repressing MLL4 target genes [77]. Genome-wide association studies and PrediXcan (a gene-based association method) for schizophrenia and bipolar disorder identified that PRMT7 was significantly correlated with schizophrenia. Further analysis showed that PRMT7 was highly expressed in the CA1 field of the hippocampus, and PRMT7^−/−^ mice displayed defects in social behaviors [78]. Recent studies further revealed that PRMT7^−/−^ CA1 neurons exhibited HCN (hyperpolarization-activated, cyclic-nucleotide-gated) channel current dysfunction, which might be attributed to the decreased HCN protein levels mediated by SHANK3 downregulation [68, 79]. These findings provide insights into the role and the underlying mechanisms of PRMT7 functioning in neuronal development.

## 4. The Roles of PRMT7 in Adipogenesis

Obesity was observed in many patients with PRMT7 mutations [70]. PRMT7 was found expressed in brown and white adipose tissues of mouse, and PRMT7^−/−^ mice at middle age developed obesity with excessive body fat accumulation [67]. Hu and colleagues used C3H10T1/2 mesenchymal cells to investigate the role of PRMT7 in adipogenic differentiation. They found that PRMT7 expression was stable during the adipogenic differentiation process. Neither PRMT7 knockdown nor PRMT7 overexpression affected lipid accumulation or adipogenic gene expression [80]. However, in a recent study, PRMT7-deleted 3T3-L1 preadipocytes or PRMT7-knocked out mouse embryonic fibroblasts displayed increased adipogenesis. In contrast, when PRMT7 was overexpressed, adipogenesis was attenuated. Further analysis discovered that PRMT7 suppressed adipogenesis by interacting with and methylating C/EBP-*β* [20, 81], which indicates a suppressive role of PRMT7 in adipogenesis.

## 5. The Roles of PRMT7 in B Cell Development

PRMT7 was also highly expressed in adult lymphoid tissues, including bone marrow and spleen, suggesting its possible roles in immunity [65]. B cells, which are derived from hematopoietic progenitor cells in bone marrow, are critical components of immunity by producing antibodies against pathogens in the human body. Deficient B cell development can cause reduced antibody production, allergy, and malignancy [82]. Therefore, Ying et al. generated B cell-specific PRMT7 conditional knockout mice (PRMT7-CKO mice) for identifying the role of PRMT7 in B cell development [65]. These PRMT7-CKO mice exhibited significant splenomegaly, and their splenic cells showed decreased mature marginal zone B cells and increased follicular B cells, which indicated that PRMT7 deficiency impaired late B cell differentiation and promoted germinal center hyperplasia [82, 83]. In addition, the expression of germinal center gene Bcl6, a master regulator of germinal center B cell program [84, 85], was repressed in PRMT7-CKO mice. The inhibition of Bcl6 expression was attributed to PRMT7 directly binding to its promoter [65]. These findings demonstrate that PRMT7 is important for the germinal center formation during B cell development in immunity through repressing Bcl6 expression.

## 6. The Roles of PRMT7 in Male Reproduction

Based on the presence of more epididymal fat in PRMT7^−/−^ mice and cryptorchidism in male patients with PRMT7 mutations [67, 70], it is expected that PRMT7 may have a function in male reproduction. Jelinic et al. discovered that PRMT7 was expressed in germ cells of newborn and adult mouse testes [18]. Moreover, PRMT7 interacted with the testis-specific factor CTCFL (CCCTC-binding factor (zinc finger protein)-like), and both were expressed during embryonic male germ cell development. Further analysis revealed that CTCFL functioned as an accessory protein for stimulating PRMT7 activity [18, 86]. A loss-of-function of PRMT7 study demonstrated that PRMT7 was determined as a critical factor for the development of mouse male germ cells during embryonic stages [87]. The size and weight of testes, the diameter of seminiferous tubules, and the number of germ cells were reduced in PRMT7 knockout male mice compared with control mice. Mechanistic analyses revealed that PRMT7 impeded male germ cell proliferation, which was possibly attributed to the TGF-*β* signaling pathway. In addition, our recent work also discovered that PRMT7 regulated the proliferation of male mouse germ cells (unpublished data). These discoveries imply that PRMT7 has important roles in male reproduction.

## 7. The Roles of PRMT7 in Cellular Stress and Senescence

Recently, to identify the interactome and potential substrates of PRMT7, Haghandish et al. performed quantitative mass spectrometry experiments and identified eIF2*α* as a novel substrate of PRMT7. They further discovered that PRMT7 regulated eIF2*α*-dependent stress granule formation in response to various cellular stresses through methylation and subsequent phosphorylation on Ser51 of eIF2*α* [88]. Moreover, when PRMT7 was inhibited or knocked out, the levels of arginine monomethylated HSP70 proteins were significantly decreased. Further, PRMT7 can give rise to the methylation of HSP70 on the location of R649 [89]. Given the discoveries that eIF2*α* as a nonhistone substrate of PRMT7 plays a functional role in cellular stress response and HSP70 family members are associated with stress response [90], it is likely that PRMT7 serves as a crucial mediator in cellular stress response pathways.

Furthermore, PRMT7 was also found to be associated with cellular senescence. Mouse embryonic fibroblasts with PRMT7 knockout underwent premature cellular senescence which was accompanied by increased expression of p16 and p21, two known cell cycle inhibitors [91, 92]. Further results revealed that PRMT7 positively interacted with GLI2 (glioma-associated oncogene 2) and methylated GLI2 to enhance GLI2-mediated sonic hedgehog signaling activity, which reportedly is a signaling associated with cellular senescence [93, 94]. Overall, these data demonstrate that PRMT7 functions to prevent cellular senescence, suggesting the potential role of PRMT7 in antiaging.

## 8. Conclusions

PRMT7 has been the subject of extensive researches over the past two decades. Genetic deficiency of PRMT7 causes a range of abnormalities in mice and humans. PRMT7^−/−^ mice showed reduced body size, shortened metatarsal bones, and declined survival rate shortly after birth, while surviving adult mice showed increased fat mass. The phenotypes of PRMT7 mutations in humans are mainly represented as SBIDDS syndrome. The common or similar phenotypes of PRMT7^−/−^ mice and humans with PRMT7 mutations demonstrated the crucial roles of PRMT7 in the development of skeletal muscle, neurons, bone, adipose tissues, and male reproduction.

**Figure 1:**
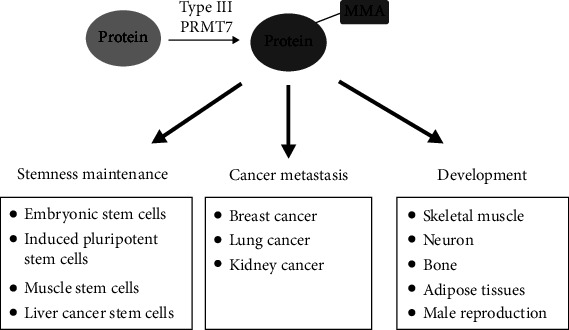
Biological functions of PRMT7 in mice and humans. PRMT7, a type III protein arginine methyltransferase, regulates the proliferation and differentiation of stem cells and promotes cancer metastasis. Deficiency of PRMT7 causes developmental defects in many systems. PRMT7: protein arginine methyltransferase 7; MMA: monomethyl arginine.

Here, we reviewed the recent research findings regarding the biological functions of PRMT7 (Figure 1). In particular, we discussed in detail the roles of PRMT7 in stem cells, including mouse ESCs, iPSCs, and MuSCs, the development of skeletal muscle and neurons, and the process of adipogenesis. Future work should also be aimed at identifying the specific substrates and functions of PRMT7, as well as the underlying molecular mechanisms. However, a few challenges prevent the identification of specific substrates of PRMT7. First, the substrates of PRMT7 and other PRMTs may overlap, and the interaction between PRMTs is complex, challenging systematic studies on PRMT7 substrates. Second, current studies drew inconsistent conclusions regarding the formation of monomethylarginine or symmetric dimethylarginine mediated by PRMT7, which may be due to contamination by other PRMTs, especially PRMT5. In addition, there is a lack of specific chemical probes and small molecule inhibitors for PRMT7, which has hindered its potential application in disease therapy. Last but not least, clarifying the roles of PRMT7 in male reproduction and the underlying mechanisms, which are the aims of our research group, will further expand our understanding of the biological roles of PRMT7 in human life and disease.

## Abbreviations

CARM1: Coactivator associated arginine methyltransferase 1
CTCFL: CCCTC-binding factor (zinc finger protein)-like
EEF2: Eukaryotic translation elongation factor 2
EMT: Epithelial-to-mesenchymal transition
ESCs: Embryonic stem cells
FAK: Focal adhesion kinase
GLI2: Glioma-associated oncogene 2
HASP5: Heat shock protein 5
HCN: Hyperpolarization-activated, cyclic-nucleotide-gated channel
hLCSCs: Human liver cancer stem cells
iPSCs: Induced pluripotent stem cells
MLL4: Mixed-lineage leukemia 4
MMP9: Matrix metalloproteinase 9
MuSCs: Muscle stem cells
NSCLC: Non-small-cell lung cancer
PRMT: Protein arginine methyltransferase
PRMT7-CKO: PRMT7 conditional knockout
SBIDDS: Short stature, brachydactyly, intellectual developmental disability, and seizures
SHANK2/3: SH3 and multiple ankyrin repeat domains 2/3.
